# (*RS*)-*N*-[(4-Chloro­phen­yl)(phen­yl)­meth­yl]­formamide

**DOI:** 10.1107/S1600536808024124

**Published:** 2008-08-06

**Authors:** Zhi-Hong Zou, Qi-Yuan Wang, Zhong-Shu Li

**Affiliations:** a Department of Pharmaceutical Engineering, College of Chemistry and Chemical Engineering, Southeast University, Nanjing 210009, People’s Republic of China; b Ordered Matter Science Research Center, College of Chemistry and Chemical Engineering, Southeast University, Nanjing 210096, People’s Republic of China

## Abstract

The racemic title compound, C_14_H_12_ClNO, contains two mol­ecules in the asymmetric unit. The dihedral angles between the phenyl and benzene rings are 84.03 (15) and 83.92 (13)°. The crystal structure involves inter­molecular N—H⋯O, C—H⋯Cl and C—H⋯O hydrogen bonds, linking mol­ecules into layers parallel to the (100) plane.

## Related literature

For related literature, see: Pflum *et al.* (2002 ▶); Wang *et al.* (2005 ▶, 2007 ▶).
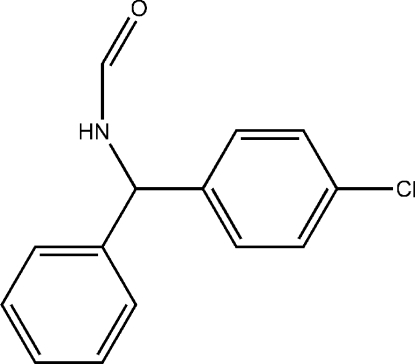


## Experimental

### 

#### Crystal data

C_14_H_12_ClNO
*M* *_r_* = 245.70
Monoclinic, 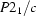

*a* = 16.830 (4) Å
*b* = 9.6318 (12) Å
*c* = 16.683 (4) Å
β = 111.538 (12)°
*V* = 2515.6 (9) Å^3^
*Z* = 8
Mo *K*α radiation
μ = 0.29 mm^−1^
*T* = 293 (2) K
0.25 × 0.20 × 0.20 mm

#### Data collection

CCD area-detector diffractometerRigaku Scxmini
Absorption correction: multi-scan (*CrystalClear*; Rigaku, 2005 ▶) *T*_min_ = 0.852, *T*_max_ = 0.940
20642 measured reflections
4421 independent reflections
2499 reflections with *I* > 2σ(*I*)
*R*_int_ = 0.079

#### Refinement

*R*[*F* ^2^ > 2σ(*F* ^2^)] = 0.074
*wR*(*F* ^2^) = 0.202
*S* = 1.06
4421 reflections
307 parameters
72 restraints
H-atom parameters constrained
Δρ_max_ = 0.73 e Å^−3^
Δρ_min_ = −0.36 e Å^−3^

### 

Data collection: *CrystalClear* (Rigaku, 2005 ▶); cell refinement: *CrystalClear*; data reduction: *CrystalClear*; program(s) used to solve structure: *SHELXS97* (Sheldrick, 2008 ▶); program(s) used to refine structure: *SHELXL97* (Sheldrick, 2008 ▶); molecular graphics: *SHELXTL/PC* (Sheldrick, 2008 ▶); software used to prepare material for publication: *SHELXTL/PC*.

## Supplementary Material

Crystal structure: contains datablocks I, global. DOI: 10.1107/S1600536808024124/rz2236sup1.cif

Structure factors: contains datablocks I. DOI: 10.1107/S1600536808024124/rz2236Isup2.hkl

Additional supplementary materials:  crystallographic information; 3D view; checkCIF report

## Figures and Tables

**Table 1:** Hydrogen-bond geometry (Å, °)

*D*—H⋯*A*	*D*—H	H⋯*A*	*D*⋯*A*	*D*—H⋯*A*
N1—H1*A*⋯O2^i^	0.86	2.02	2.877 (4)	174
N2—H2*A*⋯O1^ii^	0.86	2.16	2.901 (4)	144
C18—H18*A*⋯O2^iii^	0.93	2.54	3.368 (5)	148
C20—H20*A*⋯Cl2	0.93	2.82	3.633 (4)	146

Symmetry codes: (i) 
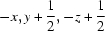
; (ii) 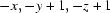; (iii) 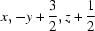.

**Fig. 1.**
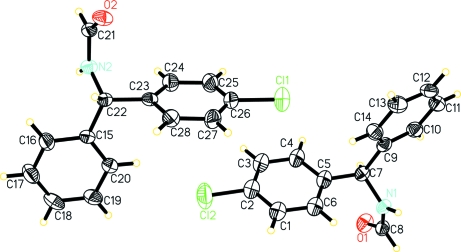
The asymmetric unit of the title compound with the atomic numbering scheme. Displacement ellipsoids are drawn at the 30% probability level.

**Fig. 2.**
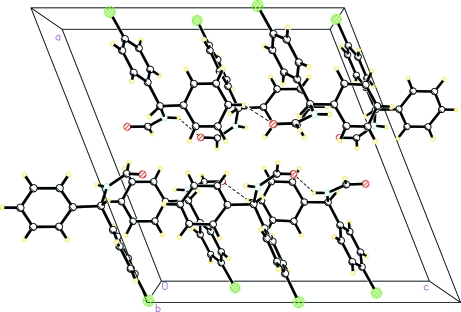
Crystal packing of the title compound viewed along the b axis. Hydrogen bonds are shown as dashed lines.

## supplementary crystallographic information

### Comment

As part of our ongoing investigations on the asymmetric synthesis, the title compound, C_14_H_12_ClNO, has been obtained as a racemic mixture and structurally characterized. The compound is the key intermediate for the synthesis of levocetirizine dihydrochloride (Pflum *et al.*, 2002; Wang *et al.*, 2007), a high effective non-sedating H_1_ receptor antagonist for the treatment of allergic diseases (Wang *et al.*, 2005). The asymmetric unit of the title compound (Fig. 1) contains two molecules. The dihedral angles formed by planes of the phenyl and benzene rings are 84.03 (15) and 83.92 (13)°. In the crystal structure (Fig. 2), intermolecular N—H···O, C—H···Cl and C—H···O hydrogen bonds (Table 1) link molecules into layers parallel to the (100) plane.

### Experimental

All chemicals used (reagent grade) were commercially available. A mixture of (4-chlorophenyl)phenylmethanone (21.67 g) and formamide (18.02 g) was stirred at 180°C for 20 h. The mixture was cooled to room temperature, and the resulting precipitate was filtered off, washed with water and dried. Colourless crystals of the title compound suitable for X-ray analysis were obtained by slow evaporation of a 60% aqueous ethanol solution.

### Refinement

All H atoms were placed in calculated positions and refined using a riding model, with C—H = 0.93–0.98 Å, N—H = 0.86 Å, and with *U*_iso_(H) = 1.2 *U*_eq_(C, N).

### Crystal data

**Table:**

C_14_H_12_ClNO	*F*_000_ = 1024
*M**_r_* = 245.70	*D*_x_ = 1.297 Mg m^−^^3^
Monoclinic, *P*2_1_/*c*	Melting point: 397(2) K
Hall symbol: -P 2ybc	Mo *K*α radiation λ = 0.71073 Å
*a* = 16.830 (4) Å	Cell parameters from 3463 reflections
*b* = 9.6318 (12) Å	θ = 2.6–27.4º
*c* = 16.683 (4) Å	µ = 0.29 mm^−^^1^
β = 111.538 (12)º	*T* = 293 (2) K
*V* = 2515.6 (9) Å^3^	Prism, colourless
*Z* = 8	0.25 × 0.20 × 0.20 mm

### Data collection

**Table:**

Rigaku Scxmini CCD area-detector diffractometer	4421 independent reflections
Radiation source: fine-focus sealed tube	2499 reflections with *I* > 2σ(*I*)
Monochromator: graphite	*R*_int_ = 0.079
Detector resolution: 8.192 pixels mm^-1^	θ_max_ = 25.0º
*T* = 293(2) K	θ_min_ = 2.9º
Thin–slice ω scans	*h* = −20→19
Absorption correction: multi-scan(CrystalClear; Rigaku, 2005)	*k* = −11→11
*T*_min_ = 0.852, *T*_max_ = 0.940	*l* = −19→19
20642 measured reflections	

### Refinement

**Table:**

Refinement on *F*^2^	Secondary atom site location: difference Fourier map
Least-squares matrix: full	Hydrogen site location: inferred from neighbouring sites
*R*[*F*^2^ > 2σ(*F*^2^)] = 0.075	H-atom parameters constrained
*wR*(*F*^2^) = 0.202	*w* = 1/[σ^2^(*F*_o_^2^) + (0.0904*P*)^2^ + 0.5905*P*] where *P* = (*F*_o_^2^ + 2*F*_c_^2^)/3
*S* = 1.06	(Δ/σ)_max_ < 0.001
4421 reflections	Δρ_max_ = 0.73 e Å^−^^3^
307 parameters	Δρ_min_ = −0.36 e Å^−^^3^
72 restraints	Extinction correction: none
Primary atom site location: structure-invariant direct methods	

### Special details

**Table:**

Geometry. All e.s.d.'s (except the e.s.d. in the dihedral angle between two l.s. planes) are estimated using the full covariance matrix. The cell e.s.d.'s are taken into account individually in the estimation of e.s.d.'s in distances, angles and torsion angles; correlations between e.s.d.'s in cell parameters are only used when they are defined by crystal symmetry. An approximate (isotropic) treatment of cell e.s.d.'s is used for estimating e.s.d.'s involving l.s. planes.
Refinement. Refinement of *F*^2^ against ALL reflections. The weighted *R*-factor *wR* and goodness of fit *S* are based on *F*^2^, conventional *R*-factors *R* are based on *F*, with *F* set to zero for negative *F*^2^. The threshold expression of *F*^2^ > σ(*F*^2^) is used only for calculating *R*-factors(gt) *etc*. and is not relevant to the choice of reflections for refinement. *R*-factors based on *F*^2^ are statistically about twice as large as those based on *F*, and *R*- factors based on ALL data will be even larger.

### Fractional atomic coordinates and isotropic or equivalent isotropic displacement parameters (Å^2^)

**Table:**

	*x*	*y*	*z*	*U*_iso_*/*U*_eq_	
Cl1	0.01712 (8)	0.62109 (17)	0.23035 (11)	0.1079 (6)	
N1	0.38686 (18)	0.7588 (3)	0.49582 (19)	0.0507 (8)	
H1A	0.3933	0.8195	0.4609	0.061*	
O1	0.43270 (19)	0.6899 (3)	0.63510 (18)	0.0748 (9)	
C1	0.1291 (3)	0.8461 (5)	0.4650 (3)	0.0799 (8)	
H1B	0.1094	0.9371	0.4604	0.096*	
C2	0.0808 (3)	0.7419 (5)	0.4757 (3)	0.0792 (8)	
C3	0.1074 (3)	0.6102 (5)	0.4809 (3)	0.0794 (7)	
H3A	0.0732	0.5389	0.4879	0.095*	
C4	0.1864 (3)	0.5799 (5)	0.4758 (3)	0.0767 (7)	
H4A	0.2042	0.4880	0.4789	0.092*	
C5	0.2386 (3)	0.6838 (5)	0.4664 (3)	0.0734 (7)	
C6	0.2087 (3)	0.8181 (5)	0.4607 (3)	0.0768 (7)	
H6A	0.2421	0.8908	0.4540	0.092*	
C7	0.3235 (2)	0.6486 (4)	0.4612 (2)	0.0495 (10)	
H7A	0.3450	0.5663	0.4972	0.059*	
C8	0.4345 (2)	0.7687 (4)	0.5785 (3)	0.0556 (10)	
H8A	0.4728	0.8424	0.5951	0.067*	
C9	0.3201 (2)	0.6125 (4)	0.3711 (2)	0.0497 (9)	
C10	0.3871 (3)	0.5419 (4)	0.3602 (3)	0.0613 (11)	
H10A	0.4330	0.5137	0.4086	0.074*	
C11	0.3875 (3)	0.5125 (4)	0.2798 (3)	0.0693 (12)	
H11A	0.4334	0.4654	0.2741	0.083*	
C12	0.3200 (3)	0.5526 (5)	0.2079 (3)	0.0680 (12)	
H12A	0.3208	0.5340	0.1535	0.082*	
C13	0.2516 (3)	0.6198 (5)	0.2156 (3)	0.0685 (12)	
H13A	0.2057	0.6455	0.1666	0.082*	
C14	0.2510 (3)	0.6495 (4)	0.2969 (3)	0.0591 (11)	
H14A	0.2042	0.6944	0.3021	0.071*	
C15	−0.3219 (2)	0.6980 (3)	0.3572 (2)	0.0422 (8)	
C16	−0.3975 (3)	0.7165 (5)	0.3709 (3)	0.0633 (12)	
H16A	−0.4486	0.6870	0.3292	0.076*	
C17	−0.3984 (3)	0.7773 (5)	0.4448 (3)	0.0763 (14)	
H17A	−0.4501	0.7885	0.4524	0.092*	
C18	−0.3243 (3)	0.8218 (5)	0.5075 (3)	0.0727 (13)	
H18A	−0.3252	0.8628	0.5576	0.087*	
C19	−0.2489 (3)	0.8050 (4)	0.4951 (3)	0.0650 (12)	
H19A	−0.1982	0.8354	0.5370	0.078*	
C20	−0.2474 (2)	0.7433 (4)	0.4207 (2)	0.0520 (10)	
H20A	−0.1955	0.7322	0.4134	0.062*	
C21	−0.3933 (2)	0.4215 (4)	0.1961 (3)	0.0481 (9)	
H21A	−0.4162	0.3342	0.1977	0.058*	
C22	−0.3231 (2)	0.6294 (4)	0.2750 (2)	0.0419 (8)	
H22A	−0.3616	0.6838	0.2268	0.050*	
C23	−0.2370 (2)	0.6242 (4)	0.2647 (2)	0.0433 (9)	
C24	−0.1850 (3)	0.5092 (4)	0.2863 (3)	0.0621 (11)	
H24A	−0.2026	0.4308	0.3077	0.074*	
C25	−0.1066 (3)	0.5083 (5)	0.2765 (3)	0.0737 (13)	
H25A	−0.0721	0.4298	0.2911	0.088*	
C26	−0.0805 (3)	0.6240 (5)	0.2453 (3)	0.0634 (12)	
C27	−0.1291 (3)	0.7407 (5)	0.2256 (3)	0.0660 (12)	
H27A	−0.1101	0.8198	0.2062	0.079*	
C28	−0.2075 (3)	0.7400 (4)	0.2349 (2)	0.0553 (10)	
H28A	−0.2413	0.8193	0.2208	0.066*	
Cl2	−0.01756 (10)	0.7788 (3)	0.48385 (13)	0.1502 (9)	
O2	−0.39511 (18)	0.4638 (3)	0.12630 (16)	0.0611 (8)	
N2	−0.36098 (19)	0.4909 (3)	0.26976 (19)	0.0489 (8)	
H2A	−0.3623	0.4534	0.3160	0.059*	

### Atomic displacement parameters (Å^2^)

**Table:**

	*U*^11^	*U*^22^	*U*^33^	*U*^12^	*U*^13^	*U*^23^
Cl1	0.0730 (8)	0.1320 (13)	0.1412 (13)	−0.0120 (8)	0.0659 (9)	−0.0164 (10)
N1	0.0546 (19)	0.051 (2)	0.0450 (18)	−0.0095 (16)	0.0168 (15)	0.0059 (16)
O1	0.079 (2)	0.092 (2)	0.0515 (17)	−0.0125 (17)	0.0221 (15)	0.0154 (17)
C1	0.0672 (14)	0.0827 (15)	0.0949 (16)	−0.0086 (13)	0.0356 (13)	−0.0070 (14)
C2	0.0665 (14)	0.0854 (15)	0.0928 (16)	−0.0105 (13)	0.0375 (13)	−0.0053 (14)
C3	0.0674 (14)	0.0848 (15)	0.0928 (15)	−0.0139 (13)	0.0374 (13)	−0.0011 (14)
C4	0.0661 (14)	0.0805 (15)	0.0910 (15)	−0.0132 (13)	0.0376 (13)	−0.0005 (14)
C5	0.0638 (14)	0.0766 (15)	0.0887 (15)	−0.0119 (12)	0.0385 (13)	−0.0025 (14)
C6	0.0653 (14)	0.0789 (15)	0.0929 (16)	−0.0101 (13)	0.0369 (13)	−0.0053 (14)
C7	0.054 (2)	0.045 (2)	0.048 (2)	−0.0030 (18)	0.0159 (18)	0.0099 (18)
C8	0.052 (2)	0.065 (3)	0.051 (2)	−0.010 (2)	0.021 (2)	−0.001 (2)
C9	0.053 (2)	0.036 (2)	0.060 (2)	−0.0040 (18)	0.0203 (19)	0.0036 (19)
C10	0.056 (3)	0.051 (2)	0.074 (3)	0.002 (2)	0.020 (2)	−0.005 (2)
C11	0.066 (3)	0.059 (3)	0.089 (3)	−0.001 (2)	0.036 (3)	−0.018 (3)
C12	0.089 (3)	0.060 (3)	0.061 (3)	−0.018 (3)	0.035 (3)	−0.023 (2)
C13	0.075 (3)	0.066 (3)	0.060 (3)	0.001 (2)	0.019 (2)	−0.002 (2)
C14	0.060 (3)	0.058 (3)	0.060 (3)	0.008 (2)	0.023 (2)	0.001 (2)
C15	0.052 (2)	0.0359 (19)	0.0396 (19)	−0.0002 (17)	0.0179 (17)	−0.0014 (16)
C16	0.054 (3)	0.080 (3)	0.059 (3)	0.003 (2)	0.025 (2)	−0.011 (2)
C17	0.076 (3)	0.092 (4)	0.072 (3)	0.015 (3)	0.041 (3)	−0.010 (3)
C18	0.107 (4)	0.066 (3)	0.053 (3)	0.021 (3)	0.039 (3)	−0.007 (2)
C19	0.077 (3)	0.066 (3)	0.049 (2)	0.006 (2)	0.019 (2)	−0.011 (2)
C20	0.057 (2)	0.051 (2)	0.049 (2)	0.0010 (19)	0.0214 (19)	−0.0039 (19)
C21	0.052 (2)	0.041 (2)	0.055 (2)	−0.0070 (18)	0.0234 (19)	−0.007 (2)
C22	0.049 (2)	0.039 (2)	0.0384 (19)	−0.0052 (17)	0.0163 (16)	−0.0016 (16)
C23	0.054 (2)	0.041 (2)	0.0353 (18)	−0.0042 (18)	0.0162 (16)	−0.0037 (17)
C24	0.064 (3)	0.047 (2)	0.083 (3)	0.004 (2)	0.037 (2)	0.005 (2)
C25	0.061 (3)	0.061 (3)	0.107 (4)	0.007 (2)	0.040 (3)	−0.006 (3)
C26	0.056 (2)	0.075 (3)	0.069 (3)	−0.013 (2)	0.035 (2)	−0.017 (3)
C27	0.069 (3)	0.074 (3)	0.066 (3)	−0.015 (3)	0.037 (2)	0.001 (2)
C28	0.065 (3)	0.047 (2)	0.060 (2)	−0.001 (2)	0.029 (2)	0.008 (2)
Cl2	0.0714 (9)	0.235 (2)	0.1627 (17)	−0.0194 (12)	0.0645 (10)	−0.0648 (16)
O2	0.086 (2)	0.0561 (17)	0.0452 (16)	−0.0077 (15)	0.0283 (15)	−0.0078 (14)
N2	0.063 (2)	0.0477 (19)	0.0403 (17)	−0.0097 (16)	0.0237 (15)	−0.0029 (15)

### Geometric parameters (Å, °)

**Table:**

Cl1—C26	1.751 (4)	C14—H14A	0.9300
N1—C8	1.320 (5)	C15—C20	1.382 (5)
N1—C7	1.464 (4)	C15—C16	1.385 (5)
N1—H1A	0.8600	C15—C22	1.514 (5)
O1—C8	1.220 (4)	C16—C17	1.369 (6)
C1—C2	1.345 (6)	C16—H16A	0.9300
C1—C6	1.394 (6)	C17—C18	1.371 (6)
C1—H1B	0.9300	C17—H17A	0.9300
C2—C3	1.337 (7)	C18—C19	1.368 (6)
C2—Cl2	1.746 (5)	C18—H18A	0.9300
C3—C4	1.394 (6)	C19—C20	1.385 (5)
C3—H3A	0.9300	C19—H19A	0.9300
C4—C5	1.378 (6)	C20—H20A	0.9300
C4—H4A	0.9300	C21—O2	1.224 (4)
C5—C6	1.378 (6)	C21—N2	1.327 (4)
C5—C7	1.502 (6)	C21—H21A	0.9300
C6—H6A	0.9300	C22—N2	1.467 (4)
C7—C9	1.523 (5)	C22—C23	1.521 (5)
C7—H7A	0.9800	C22—H22A	0.9800
C8—H8A	0.9300	C23—C24	1.375 (5)
C9—C10	1.385 (5)	C23—C28	1.385 (5)
C9—C14	1.397 (5)	C24—C25	1.387 (6)
C10—C11	1.373 (6)	C24—H24A	0.9300
C10—H10A	0.9300	C25—C26	1.368 (6)
C11—C12	1.370 (6)	C25—H25A	0.9300
C11—H11A	0.9300	C26—C27	1.358 (6)
C12—C13	1.367 (6)	C27—C28	1.385 (5)
C12—H12A	0.9300	C27—H27A	0.9300
C13—C14	1.390 (6)	C28—H28A	0.9300
C13—H13A	0.9300	N2—H2A	0.8600
			
C8—N1—C7	122.6 (3)	C20—C15—C16	117.5 (3)
C8—N1—H1A	118.7	C20—C15—C22	122.6 (3)
C7—N1—H1A	118.7	C16—C15—C22	119.8 (3)
C2—C1—C6	120.1 (5)	C17—C16—C15	121.3 (4)
C2—C1—H1B	119.9	C17—C16—H16A	119.4
C6—C1—H1B	119.9	C15—C16—H16A	119.4
C3—C2—C1	120.7 (5)	C16—C17—C18	120.9 (4)
C3—C2—Cl2	119.5 (4)	C16—C17—H17A	119.6
C1—C2—Cl2	119.7 (4)	C18—C17—H17A	119.6
C2—C3—C4	120.0 (4)	C19—C18—C17	118.8 (4)
C2—C3—H3A	120.0	C19—C18—H18A	120.6
C4—C3—H3A	120.0	C17—C18—H18A	120.6
C5—C4—C3	121.1 (5)	C18—C19—C20	120.7 (4)
C5—C4—H4A	119.4	C18—C19—H19A	119.7
C3—C4—H4A	119.4	C20—C19—H19A	119.7
C4—C5—C6	117.2 (4)	C15—C20—C19	120.9 (4)
C4—C5—C7	120.2 (4)	C15—C20—H20A	119.6
C6—C5—C7	122.6 (4)	C19—C20—H20A	119.6
C5—C6—C1	120.8 (4)	O2—C21—N2	124.9 (3)
C5—C6—H6A	119.6	O2—C21—H21A	117.6
C1—C6—H6A	119.6	N2—C21—H21A	117.6
N1—C7—C5	112.6 (3)	N2—C22—C15	108.2 (3)
N1—C7—C9	108.2 (3)	N2—C22—C23	112.0 (3)
C5—C7—C9	114.6 (3)	C15—C22—C23	114.8 (3)
N1—C7—H7A	107.0	N2—C22—H22A	107.2
C5—C7—H7A	107.0	C15—C22—H22A	107.2
C9—C7—H7A	107.0	C23—C22—H22A	107.2
O1—C8—N1	125.9 (4)	C24—C23—C28	117.7 (4)
O1—C8—H8A	117.1	C24—C23—C22	122.6 (3)
N1—C8—H8A	117.1	C28—C23—C22	119.7 (3)
C10—C9—C14	117.5 (4)	C23—C24—C25	121.1 (4)
C10—C9—C7	120.3 (3)	C23—C24—H24A	119.5
C14—C9—C7	122.2 (3)	C25—C24—H24A	119.5
C11—C10—C9	121.7 (4)	C26—C25—C24	119.4 (4)
C11—C10—H10A	119.2	C26—C25—H25A	120.3
C9—C10—H10A	119.2	C24—C25—H25A	120.3
C12—C11—C10	119.8 (4)	C27—C26—C25	121.2 (4)
C12—C11—H11A	120.1	C27—C26—Cl1	119.5 (3)
C10—C11—H11A	120.1	C25—C26—Cl1	119.4 (4)
C13—C12—C11	120.5 (4)	C26—C27—C28	118.8 (4)
C13—C12—H12A	119.8	C26—C27—H27A	120.6
C11—C12—H12A	119.8	C28—C27—H27A	120.6
C12—C13—C14	119.8 (4)	C27—C28—C23	121.8 (4)
C12—C13—H13A	120.1	C27—C28—H28A	119.1
C14—C13—H13A	120.1	C23—C28—H28A	119.1
C13—C14—C9	120.6 (4)	C21—N2—C22	122.3 (3)
C13—C14—H14A	119.7	C21—N2—H2A	118.8
C9—C14—H14A	119.7	C22—N2—H2A	118.8
			
C6—C1—C2—C3	−1.2 (8)	C20—C15—C16—C17	0.0 (6)
C6—C1—C2—Cl2	178.3 (4)	C22—C15—C16—C17	179.5 (4)
C1—C2—C3—C4	0.5 (8)	C15—C16—C17—C18	0.0 (7)
Cl2—C2—C3—C4	−179.0 (4)	C16—C17—C18—C19	0.2 (7)
C2—C3—C4—C5	0.7 (7)	C17—C18—C19—C20	−0.4 (7)
C3—C4—C5—C6	−1.1 (7)	C16—C15—C20—C19	−0.1 (6)
C3—C4—C5—C7	179.9 (4)	C22—C15—C20—C19	−179.7 (4)
C4—C5—C6—C1	0.4 (7)	C18—C19—C20—C15	0.4 (6)
C7—C5—C6—C1	179.3 (4)	C20—C15—C22—N2	121.4 (4)
C2—C1—C6—C5	0.8 (8)	C16—C15—C22—N2	−58.2 (4)
C8—N1—C7—C5	85.7 (4)	C20—C15—C22—C23	−4.5 (5)
C8—N1—C7—C9	−146.6 (3)	C16—C15—C22—C23	176.0 (3)
C4—C5—C7—N1	−149.9 (4)	N2—C22—C23—C24	−26.9 (5)
C6—C5—C7—N1	31.2 (6)	C15—C22—C23—C24	97.0 (4)
C4—C5—C7—C9	85.8 (5)	N2—C22—C23—C28	155.0 (3)
C6—C5—C7—C9	−93.1 (5)	C15—C22—C23—C28	−81.1 (4)
C7—N1—C8—O1	0.6 (6)	C28—C23—C24—C25	−1.5 (6)
N1—C7—C9—C10	71.5 (4)	C22—C23—C24—C25	−179.6 (4)
C5—C7—C9—C10	−161.9 (4)	C23—C24—C25—C26	0.3 (7)
N1—C7—C9—C14	−107.9 (4)	C24—C25—C26—C27	1.5 (7)
C5—C7—C9—C14	18.7 (5)	C24—C25—C26—Cl1	−178.3 (3)
C14—C9—C10—C11	2.0 (6)	C25—C26—C27—C28	−2.0 (7)
C7—C9—C10—C11	−177.5 (4)	Cl1—C26—C27—C28	177.8 (3)
C9—C10—C11—C12	−0.4 (7)	C26—C27—C28—C23	0.8 (6)
C10—C11—C12—C13	−1.1 (7)	C24—C23—C28—C27	0.9 (6)
C11—C12—C13—C14	1.0 (7)	C22—C23—C28—C27	179.1 (3)
C12—C13—C14—C9	0.6 (7)	O2—C21—N2—C22	0.0 (6)
C10—C9—C14—C13	−2.0 (6)	C15—C22—N2—C21	160.2 (3)
C7—C9—C14—C13	177.4 (4)	C23—C22—N2—C21	−72.3 (4)

### Hydrogen-bond geometry (Å, °)

**Table:**

*D*—H···*A*	*D*—H	H···*A*	*D*···*A*	*D*—H···*A*
N1—H1A···O2^i^	0.86	2.02	2.877 (4)	174
N2—H2A···O1^ii^	0.86	2.16	2.901 (4)	144
C18—H18A···O2^iii^	0.93	2.54	3.368 (5)	148
C20—H20A···Cl2	0.93	2.82	3.633 (4)	146

Symmetry codes: (i) −*x*, *y*+1/2, −*z*+1/2; (ii) −*x*, −*y*+1, −*z*+1; (iii) *x*, −*y*+3/2, *z*+1/2.
